# The Size Dependence of Microwave Permeability of Hollow Iron Particles

**DOI:** 10.3390/s22083086

**Published:** 2022-04-18

**Authors:** Anastasia V. Artemova, Sergey S. Maklakov, Alexey V. Osipov, Dmitriy A. Petrov, Artem O. Shiryaev, Konstantin N. Rozanov, Andrey N. Lagarkov

**Affiliations:** Institute for Theoretical and Applied Electromagnetics RAS, Moscow 125412, Russia; squirrel498@gmail.com (S.S.M.); avosipov@mail.ru (A.V.O.); dpetrov-itae@yandex.ru (D.A.P.); artemshiryaev@mail.ru (A.O.S.); k.rozanov@yandex.ru (K.N.R.); maklakov@itae.ru (A.N.L.)

**Keywords:** ultrasonic spray pyrolysis, hollow particles, ferromagnetic powder, microwave permeability, Curie temperature

## Abstract

Hollow ferromagnetic powders of iron were obtained by means of ultrasonic spray pyrolysis. A variation in the conditions of the synthesis allows for the adjustment of the mean size of the hollow iron particles. Iron powders were obtained by this technique, starting from the aqueous solution of iron nitrate of two different concentrations: 10 and 20 wt.%. This was followed by a reduction in hydrogen. An increase in the concentration of the solution increased the mean particle size from 0.6 to 1.0 microns and widened particle size distribution, but still produced hollow particles. Larger particles appeared problematic for the reduction, although admixture of iron oxides did not decrease the microwave permeability of the material. The paraffin wax-based composites filled with obtained powders demonstrated broadband magnetic loss with a complex structure for lesser particles, and single-peak absorption for particles of 1 micron. Potential applications are 5G technology, electromagnetic compatibility designs, and magnetic field sensing.

## 1. Introduction

Ferromagnetic powders of submicron size are in demand due to potential applications in magnetic data storage systems, catalysis, magnetic field sensors, biomedical treatment, and microwave absorption materials [1,2,3,4]. Diverse chemical and physical techniques for the preparation of ferromagnetic powders exist. The most common techniques are chemical vapor deposition [5], sol-gel technology [6], mechanochemistry [7], and the ultrasonic spray pyrolysis (USP) technique.

The method of ultrasonic spray pyrolysis is designed to produce powders of metals or metal oxides from aqueous solutions. This method allows for adjustment of the properties of the final product: the shape, size, and composition of particles [8,9]. The technology consists of ultrasonic atomization of a precursor solution that produces an aerosol, then drying the aerosol droplets under high temperature, followed by collecting particles on a filter. The droplet size is controlled by the frequency of ultrasonic treatment, and by the concentration of the precursor solution [8,9,10]. Heating of aerosol causes diffusion of the dissolved substance to the surface of the droplet and evaporation of the solvent. In other terms, the droplets undergo drying, thus producing spherical solid particles. Depending on the temperature and duration of the droplet being in the hot zone, different particle size, shape, and morphology is formed. Interestingly, it was reported [11], that using not a pure compound as a solvent but a mixture of two compounds (for example, water and ethanol), made it possible to change the shape of obtained particles from spheres to flakes. It is proposed that not only surface tension of the solvent affects the mechanism of particle formation, but so do differences in the evaporation rate of the components of the solvent.

The spray pyrolysis enables one to obtain powders of metal oxides [11,12,13,14] and metals [10,15,16,17] with a mean diameter from nanometers to micrometers [18]. It is possible to obtain solid or hollow metal oxide particles [10]. Metal oxides can be reduced to metals via a one-stage process, or by applying post-deposition reduction during the second stage [15] that enhances control on phase and average size formation. The one-stage process can combine USP and hydrogen flow reduction [8,9], or reducing agents can be added to the precursor solution [16]. The concentration and type of reduction agents influence not only the size and morphology of particles, but also the reduction degree of particles. Additionally, thin films [19,20] and core-shell structures can be produced by the addition of alcohol [21] or silica sol [22] to the solution in the one-step USP process.

Properties of nano- and micrometer particles may differ particularly from their bulk counterparts [23]. Shape, size, and structure as well as chemical composition have critical value for static and dynamic magnetic properties [24,25]. Considering the effect of the chemical composition of the precursor (Fe(NO_3_)_3_, FeCl, etc.) on structure, as well as size- and structure-dependence of magnetic properties [16,21] of the final product, the spray pyrolysis technique offers a versatile possibility to control magnetic properties and electromagnetic parameters of functional materials. Developing new methods to effectively produce magnetic particles with complex structures and desired magnetic properties is still a challenge, especially in the vastly expanding field of magnetic sensing for biology and health applications.

Here, the USP technique followed by hydrogen reduction was applied to obtain iron particles with hollow structures. Variations in the solution of the precursor, Fe(NO_3_)_3_, produced powders with mean particle sizes of 0.6 and 1 μm. The difference in the particle size changed magnetic loss peak quality factor as well as imaginary permittivity of the composites based on paraffin wax matrix.

## 2. Materials and Methods

### 2.1. Ultrasonic Spray Pyrolysis and Reduction with Hydrogen

The iron powders were obtained using a two-stage synthesis similar to what was previously reported [17]. In the first stage, two aqueous solutions of iron (III) nitrate nonahydrate, Fe(NO_3_)_3_·9H_2_O with concentrations of 10 and 20 wt.% (0.5 and 1 M, respectively) were prepared. An aerosol was obtained from these solutions using an ultrasonic dispenser (Timberk, China). Then, the aerosol was transferred through a tube furnace Nabertherm RT 50/250/13 (Nabertherm GmbH, Lilienthal, Germany) with airflow (16 L/min). The stainless steel tube 1000 mm (Ø 50 mm) in length was used as reactor, and the length of the heat zone in the furnace was about 30 cm. As a result of brief heat treatment at 1000 °C and chemical decomposition of Fe(NO_3_)_3_, a Fe_2_O_3_ oxide was formed and collected on a filter. In the second stage, iron oxide powders were reduced to metal in a tube furnace, Carbolite HZS 12/600 (Carbolite Gero, Neuhausen, Germany), at 450 °C in a flow of hydrogen. Metal powders were passivated in nitrogen for 12 h at room temperature. Hereafter, samples which were obtained starting from 10 wt.% solution and 20 wt.% solution are denoted as Fe^10%^ and Fe^20%^, respectively.

### 2.2. Characterization

The chemical phase composition was studied through the X-ray diffraction (XRD) method using a Difray 401 diffractometer (JSC Scientific instruments, Saint Petersburg, Russia) with Cr K_α_ radiation (λ = 0.229 nm). Bragg–Brentano geometry was applied for the measurements from 14 to 140 degrees, 2θ. The detector was curved and position-sensitive. The XRD pattern was used for the semiquantitative phase analysis and evaluation of the average size of the crystallites.

Scanning electron microscope (SEM) JEOL JCM-7000 (JEOL, Tokyo, Japan) equipped with the device for energy-dispersive X-ray (EDX) analysis was applied for the study of morphology and to estimate particle size distribution of the powders. To study the size distribution of particles, diameters of approximately 1000 particles were measured from the SEM images. The EDX analysis was measured from an area of 100 × 100 μm, uniformly covered with particles; measurements were average when studying five different areas.

The investigation of the thermal stability of metal powders from 30 to 1000 °C with a heating rate of 10 K/min in Ar and air flows was carried out using synchronous thermal analysis (STA) instrument Netzsch STA 449 F3 Jupiter (Netzsch, Selb, Germany). STA stands for simultaneous measurement of thermogravimetric (TG) and differential scanning calorimetry (DSC) curves. A special setup, designed by Netzsch to measure the Curie temperature, was used. For this purpose, external magnets were fastened onto the furnace of the STA device. At the temperature of the Curie transition, the ferromagnet lost magnetic interaction with these magnets. At this moment, the weight sensor detected an abrupt change of mass, and the thermal sensor detected a weak peak that was caused by the phase transition. The Curie temperature was defined by step-like mass change, measured in Ar flow.

The magnetic hysteresis at room temperature was measured using the vibrating sample magnetometer (VSM) in a magnetic field of ±15 kOe. The complex microwave permeability (*μ*′ + *i* × *μ*″) and permittivity (*ε*′ + *i* × *ε*″) were measured from the wax-based composites with filler fractions of 33, 50, and 66 wt.% in a standard 7/3 coaxial airline with an HP 8720 vector network analyzer (Keysight, Santa Rosa, CA, USA) by the Nicolson–Ross–Weir method [26,27] in the frequency range of 0.1–30 GHz. Higher-order modes in coaxial waveguide were accounted, following previously reported procedures [28].

## 3. Results and Discussion

### 3.1. X-ray Difraction Analysis

**Figure 1:**
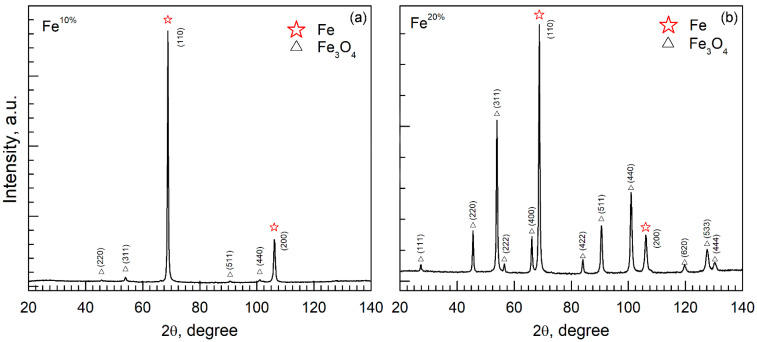
X-ray diffraction measured from the (**a**) Fe^10%^ and (**b**) Fe ^20%^ samples.

The XRD phase analysis demonstrated that the obtained powders consisted of α-Fe and Fe_3_O_4_ phases (Figure 1, Table 1). The positions of reflections of α-Fe in obtained samples (110) 68.78° 2θ and (200) 106.03° 2θ coincided well with the tabular values (ICDD No. 60696), as did the lattice constant *a*, which is 2.866 Å, both here and in the ICDD card No. 60696. For Fe_3_O_4_, the tabular value for constant *a* varies from 8.33 [29] to 8.432 Å [30]. Here, the lattice constant was within this range. The crystallite size of powders which was calculated using the Scherrer formula [31] was approximately 30 nm for all phases.

The reference intensity ratio (RIR) method [32] with corundum as a reference standard for semiquantitative phase analysis was applied. For estimation, the following reference value for α-Fe, Fe_3_O_4_ is 10.77 (ICDD No. 60696), and 4.81 (ICDD No. 11111) were used, respectively. The estimation of relative metal to oxide (ω^Fe^, ω^oxide^) content in obtained samples is provided in Table 1. Whereas both powders (Fe^10%^ and Fe^20%^) were reduced under identical conditions (H_2_ flow, 450 °C), a considerable lack of metal phase in Fe^20%^ was observed. Shatrova et al. [33] examined the H_2_ reduction of Co powder prepared through the spray pyrolysis technique at different temperatures, and demonstrated that the purity of the metal depended on the reduction temperature. In addition, particles of different sizes (and with different wall thickness, with respect to hollow structures) may react differently to passivation with the nitrogen stage. Here, identical reduction and passivation conditions were chosen intentionally for direct comparison. Further studies involving higher reduction temperatures are required.

### 3.2. SEM and EDX Analyses

**Figure 2:**
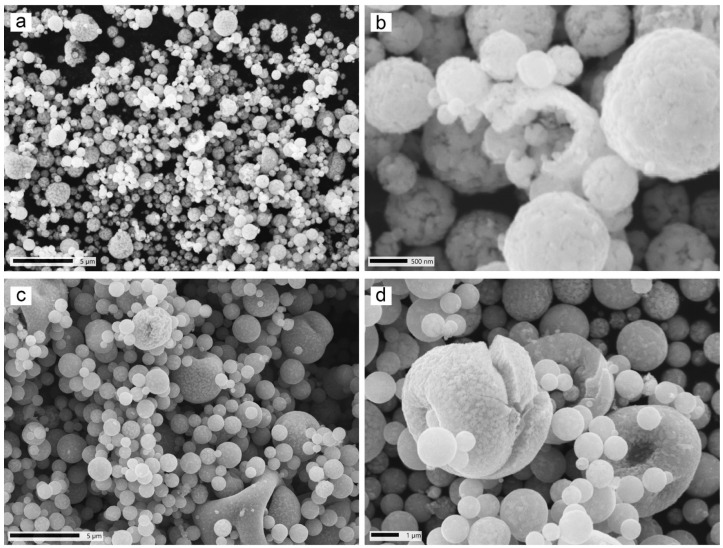
SEM-microphotographs of obtained iron powders (**a**,**b**) Fe^10%^ and (**c**,**d**) Fe^20%^.

The prepared samples possessed a spherical shape (Figure 2), which is typical for spray pyrolysis [18]. The obtained particles demonstrated porous and inhomogeneous surfaces. “Broken” particles confirmed the hollow structure.

**Figure 3:**
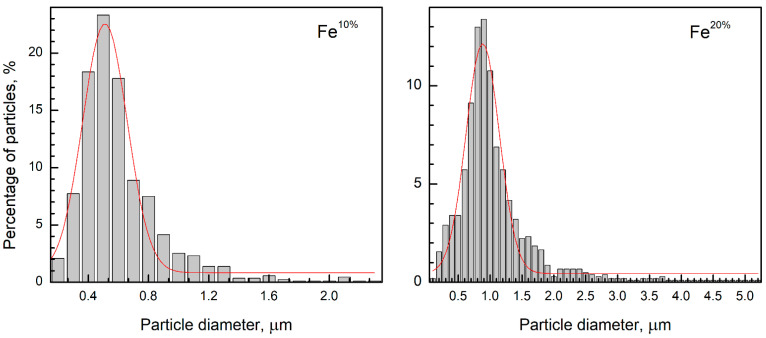
Particle size distribution, measured from the SEM images, of the Fe^10%^ and Fe^20%^ powders, where red line is Gaussian function.

An increase in the precursor solution concentration increased the mean particle size (see Figure 2a vs. Figure 2c), as well as width of particle size distribution (Figure 3). The average sizes of particles of Fe^10%^ and Fe^20%^ were estimated at 0.6 and 1 µm, respectively. Full width at half maximum is increase from 0.35 to 0.62, respectively. The thicknesses of the walls of the particle were approximated at 200 (Fe^10%^) and 500 nm (see Figure 2b,d), using SEM microphotographs. The size of particles and thickness of the walls both were larger than the crystallite size. SEM images also illustrated a grain structure, with grains around 100 nm. However, since these images do not allow for a clear distinction between the real grain size, surface morphology, and surface roughness, the X-ray data is more accurate.

Local EDX analysis illustrated the presence of iron and oxygen. The oxygen to iron ratio was estimated at 10/90 (Fe^10%^) and 30/70 at. % (Fe^20%^).

### 3.3. STA Analysis

**Figure 4:**
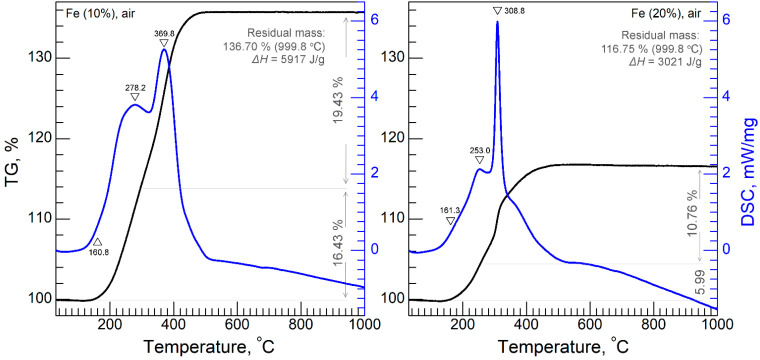
STA analysis of Fe^10%^ and Fe^20%^ powders in air.

Thermal analysis in airflow was performed for Fe^10%^ and Fe^20%^ (Figure 4). The mass gain occurred in at least two steps for both samples. These steps were accompanied by exothermic maxima in DSC curves. Step-like oxidation is typical for iron powders (see Figure 3 from [34], for example). According to [34,35], iron oxidation occurs in these steps: Fe → FeO → Fe_3_O_4_ → Fe_2_O_3_. However, direct observation of the intermediate phases is not found in the literature, partially because these oxidation steps intermix with each other, and because no technique to stop oxidation at an intermediate stage seems to be known. The total mass gain is important, and is a characteristic of the metal’s purity. For comparison, oxidation of the commercial carbonyl iron (CI, ≥ 97.0 wt. % Fe) [36] produces a weight increase of 38%. Additional measurements during this study illustrated a weight increase of 38.8% at 1000 °C for CI, which was in a good agreement with the ref. [36] data.

Considering problem of intermediate oxidation stages, simple numeric estimation of mass gain during oxidation of Fe_3_O_4_ to Fe_2_O_3_, produces 3.2–3.3 wt.%, which was confirmed in [37]. The measured weight increase during formation of the highest-oxidation state of iron was far from 3%—it was actually 10–20%. This implies that the oxidation mechanism of the obtained samples is far more complex than that proposed in [34,35]. The temperature where maximum weight was achieved was 500 °C. The formation of Fe_2_O_3_ above 500 °C was confirmed with the XRD analysis.

The total mass gains for Fe^10%^ and Fe^20%^ were 36.7 and 16.8%, respectively. Comparing total mass gain between samples Fe^10%^ and Fe^20%^, it can be concluded that the Fe^10%^ sample was of higher purity than Fe^20%^.

The total released energy for Fe^10%^ (Δ*H* = 5917 J/g) was twice as much as that of Fe^20%^ (Δ*H* = 3021 J/g). Although there is data suggesting that an increase in the particle size of iron from 1–3 to 4–10 µm [35] increases enthalpy of oxidation from 4.8 to 5.4 J/g, just the opposite effect was observed in this study. This was probably due to different purity of samples Fe^10%^ and Fe^20%^, and because of the much lower particle size than that of powders studied in [35]. The onset temperature (~160 °C) of the exothermic process was equal for both powders.

**Figure A1:**
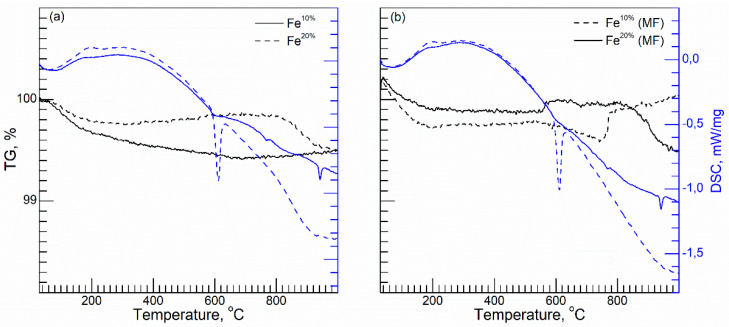
TG and DSC graphs of Fe^10%^ and Fe^20%^ powders in Ar (**a**) without magnetic field (MF) and (**b**) with external MF.

Measurements in an inert atmosphere for Fe^10%^ and Fe^20%^ were carried out (Figure A1a). Insignificant mass loss of 1% was measured for both samples. This was due to emission of nitrogen and carbon admixtures, and coincided with the loss of 2% reported in [38]. Several weak endothermic minima observed in the DSC curve were probably related to melting or sintering of particles.

The Curie temperature (T_C_) of prepared powders was defined by a step-like mass increase on the TG curve [39], accompanied with an exothermic DSC peak (Figure A1b). The T_C_ for Fe^10%^ powder corresponded to bulk iron that is about 770 °C (1043 K) [40]. The T_C_ for Fe^20%^ was lower by 30% of this value (564 °C) (see Figure A1 in Appendix A). The Curie temperature for Fe_3_O_4_ is known to be 440 °C (713 K) [41]. Since the Curie temperature is dependent on the Fe purity [39], the Fe^20%^ sample may be concluded to possess lower purity than Fe^10%^.

### 3.4. Magnetic Properties

**Figure 5:**
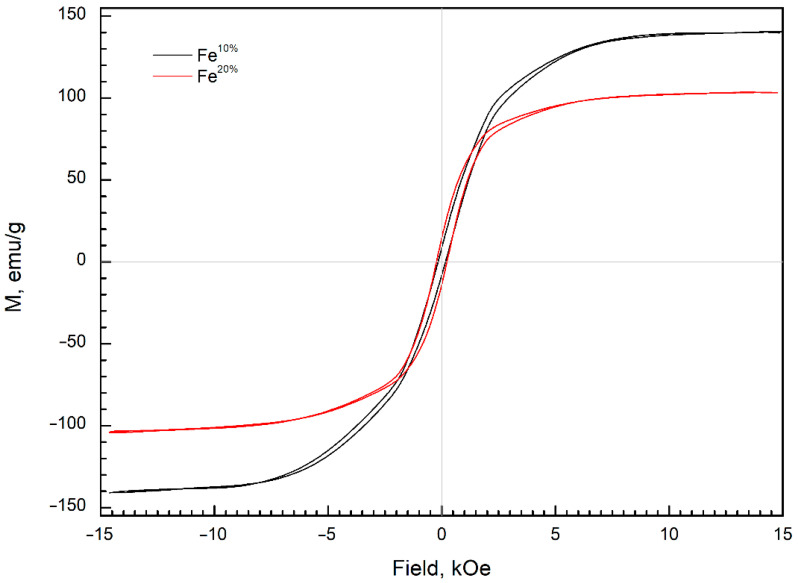
Hysteresis loops, measured from composites based on paraffin wax and filled with Fe^10%^ and Fe^20%^ powders.

Magnetic hysteresis loops of the composites containing 66 wt.% of obtained powders are displayed in Figure 5.

Considering the concentration of the powders in composites, the saturation magnetizations (*M_S_*) of the Fe^10%^ and Fe^20%^ powders were 140 and 103 emu/g, respectively (Table 1). The saturation magnetization of the carbonyl iron (CI) ranges from 175 to 188 emu/g [36,42], depending on the particle size [43]. The *M_S_* of Fe^10%^ and Fe^20%^ were lower than the aforementioned range for CI, but higher than that reported for Fe_3_O_4_ (40 and 92 emu/g) [24,44].

The *H_C_* for the Fe^10%^ and Fe^20%^ particles were 160 and 225 Oe, respectively (Table 1). The high value of the coercive force *H_C_* and moderate values of *M_S_* were probably related to the presence of the oxide phase [17].

Chemical purity of iron in both Fe^10%^ and Fe^20%^ samples can be estimated only simply. Comparing weight gain during oxidation, relative oxygen content in EDX, and XRD intensities and saturation magnetization between both samples, it appears clear that the Fe^10%^ contained a larger metal fraction than the Fe^20%^ sample.

### 3.5. Microwave Measurement of Composites

**Figure 6:**
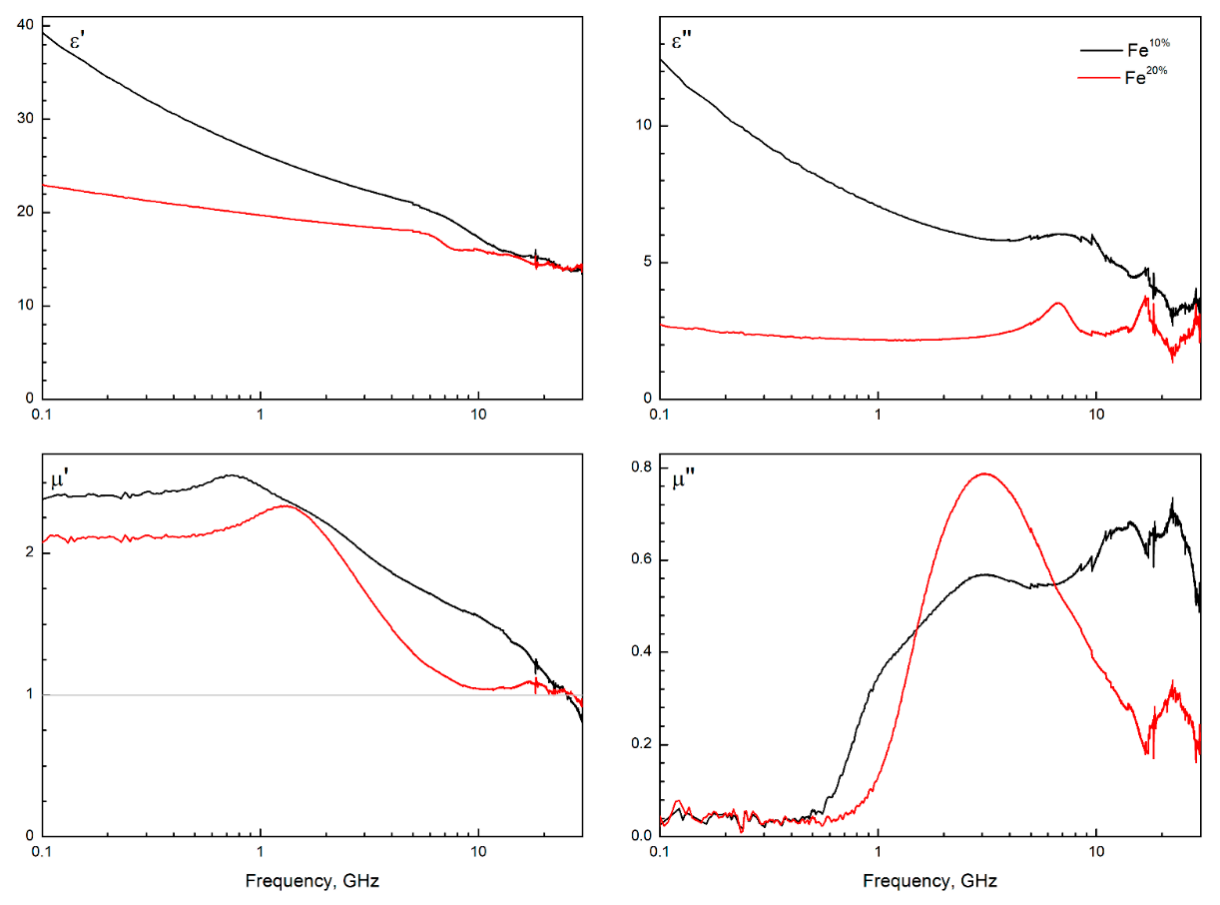
The measured frequency dependences of microwave permeability (*µ*′ + *i* × *µ*″) and permittivity (*ε*′ + *i* × *ε*″) of the composites comprising powders Fe^10%^ or Fe^20%^, and paraffin wax matrix. The curves are normalized by weight ratio 3:1 between metallic powder (Fe^10%^, Fe^20%^) and wax matrix.

The measured frequency dependencies of complex microwave characteristics of composite with 66 wt.% powder concentration are illustrated in Figure 6. An increase in the concentration (from 33 to 66 wt.%) of the fraction leads to a linear increase in the amplitude of the imaginary part of permeability.

Both powders demonstrated magnetic loss of high amplitude. Changes in size and structure of particles significantly changed the structure of the magnetic loss peak. The imaginary part of permeability of the composite filled with Fe^10%^ powder demonstrated a broad peak with a complex structure. Concurrently, composite filled with Fe^20%^ powder demonstrated a narrower peak with a single loss maximum. Both powders produced composites with low magnetic loss below 0.5 GHz, which is unusual for spherical iron (see carbonyl iron studies, [1,45]). This makes the samples promising materials for application in many areas, one being antennas.

Composite filled with Fe^10%^ demonstrated significantly higher imaginary permittivity, which was due to agglomeration of particles in the composite structure and therefore increased percolation conductivity. High saturation magnetization and low coercivity, as well as high complex permeability, illustrated that iron particles were micro-sized (not nano-sized). Also, imaginary permittivity was substantially lower for the sample with increased oxide concentration. This probably meant that contact conductivity was significantly suppressed with an increase in oxide phase concentration. In simpler terms, the oxide phase was predominantly on the surface of larger iron particles. Importantly, in a series of composites (33, 50, and 66 wt.% of a filler), the structure of the frequency dispersion and the position of the magnetic loss peak in imaginary permeability depended neither on filler concentration nor on the conductivity of certain samples.

In dynamic magnetism, a quantitative parameter that describes the overall magnetic performance is Acher’s parameter *K_A_*. This parameter was calculated following [46]:

(1) $$K_{A}=\frac{1}{kp{\left(\gamma 4\pi M_{s}\right)}^{2}}\times \frac{2}{\pi }\times \int_{f_{1}}^{f_{2}}\mu^{\prime \prime }\left(f\right)fdf$$

where *p* is the volume fraction of inclusions and *k* is a randomization factor, *γ* ≈ 3 GHz/kOe, and *f*_1_ to *f*_2_ is the frequency range of measurement.

The physical meaning of this parameter is how many of all the magnetic moments in the sample are involved in a precession that is forced by the incident microwave magnetic field. The maximum value is 1 and can only be observed in thin films. Spherical particles can possess much lower values.

In this study, the *K_A_* parameter was estimated at 0.09 for composites based on Fe^10%^ powder and 0.05 for Fe^20%^. This suggested that the Fe^10%^-based composites were more effective in absorbing electromagnetic energy microwaves than the Fe^20%^-based composites. The *K_A_* value, close to 0.1, was high [47] enough to signify those magnetic moments which were aligned along the shell of particles. This indirectly confirmed the hollow structure of the particles.

Several studies are devoted to resonances in hollow spherical particles; these three studies, [23,24,48], are worth mentioning. In conclusion, not only hollowness of magnetic particles excites resonances besides ferromagnetic, but the very distribution of particle size sophisticates the structure of magnetic loss. Considering this and the calculated *_I_K_a_* value, the Fe^10%^ sample appeared to possess higher hollowness than Fe^10%^, which increased the potential electromagnetic performance of the former.

### 3.6. Hollow or Not

The problem with the analysis of the hollowness of the obtained particles is that no direct and reliable method to do so exists. In this study, there were three signals that illustrated the hollow structure of the particles. Firstly, shatters of the hollow shells that can be seen in Figure 1b,d, as well as particles with the structure of a deflated balloon, illustrated that there are particles with a hollow structure in both samples.

The second signal is the low density of the samples. The density of wax-based composites was lower than that can be expected from composites filled with dense iron. Moreover, using the measured weights of the filler, matrix, and composite, and applying density of the wax (2.2 g/cm^3^ [2]), and effective density of the composite (calculated independently from a known volume of sample), the mean thickness of the walls of hollow particles of both powders was estimated. This can be done by considering the composite as a three-component mixture: the wax, iron, and voids filled with air. The density of inclusions was defined for each filler, then the proportion of voids was calculated. The thickness of the wall was estimated assuming that all of the particles possess the mean size (0.6 μm for Fe^10%^ and 1.0 μm for Fe^20%^). Following this procedure, the thickness of the walls of hollow particles was estimated at about 0.125 μm for both the Fe^10%^ and Fe^20%^ powders. This value was approximately in accordance with the electron microscopy data. The third ‘signal’ is that the frequency dispersion of complex permeability demonstrated features that may be interpreted as attributes of thin magnetic layers.

The formation of dense or hollow particles depends on the rate ratio of drying and precursor decomposition steps. Therefore, the temperature regime and the gas flow rate should be optimized to obtain the predominant amount of one of these two types. To further enhance this synthetic technique, additional studies are required.

## 4. Conclusions

Ferromagnetic hollow powders with the mean particle sizes of 0.6 and 1 μm were synthesized from iron nitrate solution with concentrations of 10 and 20 wt.%, respectively, by ultrasonic spray pyrolysis and reduction in hydrogen. According to XRD analysis, the impurity content (residual iron oxide) in the Fe^20%^ sample was higher than in the Fe^10%^ sample. This means that magnetic interaction between iron particles was negligible.

The weight increases during the oxidation of Fe^10%^ and Fe^20%^ in the air were 36.7 and 16.8 %, respectively. The experimentally determined Curie temperature was 770 °C for Fe^10%^, which corresponded to the tabular value for iron, and 564 °C for Fe^20%^. The difference between the values of released energy and weight increase during oxidation, as well as the Curie temperature of prepared iron powders, were caused by the presence of iron oxide in the Fe^20%^, and by the differences in the sizes of the particles.

The saturation magnetization of the Fe^10%^ and Fe^20%^ samples were 140 and 103 emu/g, respectively. The coercivity of the powder particles was moderately high (160 and 225 Oe) and was typical for powders prepared by spray pyrolysis. Both powders demonstrated low magnetic losses below 0.5 GHz. The Fe^10%^-based samples with the paraffin wax matrix demonstrated a broad spectrum of frequency dispersion of imaginary permeability with a complex pattern. At the same time, Fe^20%^-based composites demonstrate a single-peak curve. The prepared material can be applied in 5G technologies and in solutions for electromagnetic compatibility. Considering small particle size, high microwave permeability, high saturation magnetization, and potentially high biocompatibility, the obtained powders may be applied in biosensors, based on magnetic field sensing.

## Figures and Tables

**Table 1:** XRD data, where *a*—lattice constant, CS—crystallite size, ω^Fe^ and ω^oxide^—mass fraction of iron and Fe_3_O_4_ oxide in each sample, calculated by means of the RIR method, *Ms*—saturation magnetization, *Hc*—coercivity.

Sample	Phases	*a*, Å	CS, nm	ω^Fe^, wt.%	ω^oxide^, wt.%	*Ms*, emu/g	*Hc*, Oe
Fe^10%^	α-Fe (#60696)	2866	37	95	5	140	160
	Fe3O4 (#11111)	8371	23				
Fe^20%^	α-Fe (#60696)	2866	29	30	70	103	225
	Fe3O4 (#11111)	8414	29				

## Data Availability

Data is contained within the article.
